# Novel m.15434C>A (p.230L>I) Mitochondrial Cytb Gene Missense Mutation Associated with Dilated Cardiomyopathy

**DOI:** 10.5402/2012/251723

**Published:** 2012-07-03

**Authors:** Sinda Zarrouk Mahjoub, Sounira Mehri, Fatma Ourda, Josef Finsterer, Saïda Ben Arab

**Affiliations:** ^1^Genetics Laboratory and Research Unit of Genetics Epidemiology and Molecular, Faculty of Medicine of Tunis, Tunis 1007, Tunisia; ^2^Biochemistry Laboratory and Research Unit of Human Nutrition & Metabolic Disorders, Faculty of Medicine of Monastir, Monastir 5000, Tunisia; ^3^Services des Explorations Fonctionnelles Cardiologiques, Hôpital La Rabta de Tunis, Tunisia; ^4^Krankenanstalt Rudolfstiftung, Danube University Krems, Postfach 20, 1180 Vienna, Austria

## Abstract

*Background*. Previously it has been shown that various types of hypertrophic and dilative cardiomyopathy (hCMP, dCMP) can be attributed to disturbed mitochondrial oxidative energy metabolism. Several studies described mutations in mitochondrial DNA-located genes encoding for subunits of respiratory chain complexes, including the cytochrome b gene (MT-CYB), causing CMPs. *Methods and Results*. In the present study the MT-CYB gene was analysed in 30 patients with hCMP, 40 patients with dCMP, and 50 controls for alterations. Altogether, 27 MT-CYB variants were detected. Twenty-four of them were single nucleotide polymorphisms defining common haplogroups. The variant m.15434C>A was found in a single patient with severe dCMP and assessed as novel mutation, since it was not found in healthy controls or available data sets, and was nonhaplogroup associated with Phylotree. This variant altered an amino acid (L230I) with a high interspecific amino acid conservation index (CI = 97.7%) indicative of the functional importance of the residue. *Conclusions*. Though the L230I mutation seems to play a causative role for dCMP, prospective studies on yeast or transgenic mice models with defined mutation are warranted to study the pathogenetic impact of this mutation.

## 1. Introduction

Mitochondria play a critical role in both life and death of cardiomyocytes. In healthy cells, their primary function is to meet the high energy demand of the beating heart by providing ATP through oxidative phosphorylation. Mitochondria occupy a large portion of each myocyte and are located between the myofibrils or right below the sarcolemma. The strategic positioning and abundance of mitochondria ensure a highly efficient localized ATP delivery system to support contraction, metabolism, and ion homeostasis [1]. Mitochondrial disorders (MIDs) leading to myocardial disease show a strong age-dependent clinical heterogeneity [2]. Cardiac manifestations of MIDs include arrhythmias and cardiomyopathy (CMP, hypertrophic, (hCMP), dilated (dCMP), restrictive (rCMP), histiocytoid CMP, and noncompaction) [3, 4]. Mitochondria contain their own DNA (mitochondrial DNA (mtDNA)) which is a circular, 16 569 base sequence with 37 genes that encodes for 13 subunits of the respiratory chain complexes, 22 transfer RNAs, and 2 ribosomal RNAs [5]. Mutations in mtDNA-encoded genes influence the production of reactive oxygen species in mice [6] and have been implicated in a large number of human and murine diseases [7]. Some amino acid changes may also improve aerobic capacity and adaptation to new thermal environments [8]. One of the mtDNA located genes associated with cardiac disease is MT-CYB encoding for cytochrome-b. Base substitutions in the human MT-CYB gene are associated with a wide spectrum of CMPs [9, 10] or exercise intolerance [11–14]. MT-CYB is an extremely conserved protein, reflecting its fundamental role in energy production in mitochondria. It catalyses the reversible electron transfer from ubiquinol to cytochrome c coupled to proton translocation (Q-cycle) [15]. The aim of the present study was to analyse the MT-CYB gene from CMP patients and controls and to identify potential pathogenic mutations associated with hCMP or dCMP phenotypes. 

## 2. Materials and Methods 

### 2.1. Selection of Patients

Blood samples were collected from 70 patients with CMPs who originated from the area of Tunis and were recruited from the department of cardiology in the public teaching Hospital la Rabta Tunis, Tunisia. Age at onset of the disease varied from adolescence to adulthood. They comprised 40 patients with dCMP with a mean age of 39.3 ± 15.2 years and 30 patients with hCMP with a mean age of 41.87 ± 19.13 years (Table 1). All patients had angiographically normal coronary arteries and were not affected by other cardiac or systemic disease. CMP was diagnosed according to the WHO/ISFC criteria [16]. dCMP was diagnosed in case of left ventricular dilatation (LVEDD > 55 mm) and reduced ejection fraction. hCMP was diagnosed if there was myocardial thickening >14 mm on echocardiography (Table 1). Blood samples were collected with informed consent also from 50 unrelated healthy controls originating from the same area as the patients without clinical evidence or a family history of CMP. 

### 2.2. MT-Cytochrome b Gene Analysis

**Figure 1:**
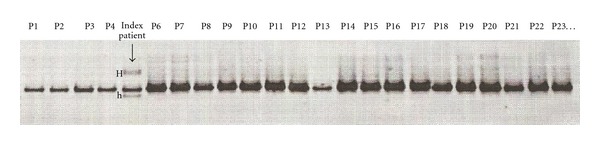
DGGE investigations illustrating the detection of heteroplasmic and homoplasmic sequence variations of the mitochondrial cytochrome b gene. The arrow shows destabilizing sequence variations in a heteroplasmic state in the index patient (P5) who presented with dCMP (H: homoduplexes, h: heteroduplex) and homoplasmic patterns in the other patients with dCMP (P1 to P4 and P6 to 23).

Total DNA extracted from lymphocytes (according to conventional methods) was submitted to polymerase chain reaction (PCR) amplification, by using two pairs of specific primers designed from the genomic sequence of the mitochondrial MT-CYB gene (14747–15887). Forward primers and reverse primers (5′-3′) were as follows: pair 1 (14667–14687 and 15289–15269) and pair 2 (15189–15209 and 15941–15921). Amplification was carried out in a total volume of 50 *μ*L containing 100 ng of total DNA, 10 pmol of each primer, 4 *μ*L MgCl_2_, 5 *μ*L dNTP, and 0.2 *μ*L Taq polymerase. The amplification conditions included 3 min at 94°C, 30 cycles of 30 s at 94°C, 60 s at 52°C, and a final extension of 10 min at 72°C terminated the PCR. The PCR products were checked by a 1.5% agarose gel electrophoresis and an ethidium bromide staining. After amplification, the PCR product was subjected to denaturing gradient gel electrophoresis (DGGE). It was run on a Tris-EDTA-acetate buffer (TEA: Tris 40 mM; Na-acetate 20 mM; EDTA 1 mM) at 60°C, using a 6.5% polyacrylamide gel containing a gradient of denaturing agents (60% denaturant = 4.2 M and 24% formamide). Gels were stained with ethidium bromide and examined under ultraviolet light, and images were saved electronically (Figure 1).

### 2.3. Direct Sequencing of PCR-Amplified Fragments

Direct sequencing of the PCR fragments was performed using an ABI 3730 automated DNA sequencer (Applied Biosystem) by using a BigDye Terminator V.3.1 cycle sequencing kit (applied 4337455 (100 reactions)) according to the manufacturer's recommendations. MT-CYB gene sequences from patients and controls were compared with the mtDNA reference, the revised Cambridge Reference Sequence (rCRS; NC_012920) [17]. 

### 2.4. Software and Databases

We used the tool PolyPhen-2 (http://genetics.bwh.harvard.edu/pph2/) [18] for predicting the damaging effect of a missense mutation. The tool PolyPhen-2 compiles two pairs of datasets, the HumDiv and the UniProt. The conservation index proposed by Ruiz-Pesini et al. [19] was computed by using MitoTool (http://www.mitotool.org/) [20]. A total of 43 primate species were considered and a conservation index (CI) of 0.744 meant that 74.4% of 43 primate species had the wild-type allele in common with the human sequence (GenBank Accession number NC_012920). The presence of the mutation as a haplogroup-specific variant was scored relative to the available global mtDNA phylogenetic tree by using Phylotree (http://phylotree.org/tree/main.htm; [21]).

## 3. Results

We sequenced the MT-CYB gene in 70 patients with CMPs and 50 controls and identified a total of 27 variants in the patients' group (Tables 2 and 3). In order to distinguish between polymorphisms and potentially deleterious mutations, we used indirect criteria, such as the position of the amino acid replacement in the protein sequence, the physicochemical properties of the amino acids involved (acidic, basic, or hydrophobic), the evolutionary conservation sequence, and the haplogroup association. Because of normal ethnic/geographic-associated haplogroup variation, however, interpretation of a mutation as pathogenic was difficult. Among the 27 variants found, one was a novel (m.15434C>A) mutation and the remaining 10 variants were already known as nonsynonymous mutations (amino acid change) associated with MIDs (Table 2, Figure 1). The patient carrying the novel MT-CYB mutation was a 24 yo male who presented with palpitations, tachycardia, a gallop rhythm, andpulmonary rales. Echocardiography revealed dilation of the left ventricle (end-diastolic LV diameter: 62 mm) and reduced left ventricular systolic function (fractional shorting: 28%, ejection fraction: 48%). In addition to dCMP with heart failure he did not have other phenotypic features of a MID. Among the previously reported nonsynonymous mutations two (m.14766T>C and m.15326A>G) were frequently encountered in our patients suffering from dCMP and hCMP with a low CI and a high frequency (>5%) and eight were rare alleles (m.14769A>G, m.14798T>C, m.14927A>G, m.15257G>A, m.15314G>A, m.15452C>A, m.15803G>A, m.15884G>C) and were classified as polymorphisms, reported in association with CMPs or other human diseases (Table 2). Sixteen variants were interpreted as synonymous mutations (no amino acid change), and were already reported as single nucleotide polymorphisms in various databases (Table 3). The m.15434C>A mutation was not detected in 50 controls as mentioned in Tables 2 and 3.

## 4. Discussion

It is well known that mutations in the MT-CYB gene are often sporadic and arise during embryogenesis, affecting a limited number of cells and resulting in tissue-specific phenotypes [22, 23]. In case MIDs exclusively affect the heart, mitochondrial hCMP or mitochondrial dCMP may be clinically indistinguishable from CMPs of other cause [3, 24]. The diagnosis of mitochondrial CMPs may be established by mtDNA sequence analysis for known pathogenic mtDNA mutations. Reviewing published literature, we detected eight nonsynonymous mutations distributed across the MT-CYB and the majority were rare (allele frequency < 5%). The amino acid change induced by these eight mutations was considered to be mild because either the modified amino acid or an amino acid with similar physicochemical characteristics was commonly encountered at that position in other animal species variants and defined common haplogroups. One of the eleven non-synonymous mutations was novel and was detected in a patient with severe dCMP. The novel m.15434C>A variant could not be found in 50 healthy controls, among confirmed variants of MID in MITOMAP: a Human Mitochondrial Genome Database (http://www.mitomap.org/, 2011) in previous studies on CMPs, or in human mitochondrial genome databases (http://www.genpat.uu.se/mtDB/). The variant was deemed pathogenic on the basis of the following findings: (1) the m.15434C>A mutation resulted in the substitution of a cytosine for an adenine residue at position 230 of MT-CYB, suggesting that it may have resulted from oxidative damage since it could not be inherited [25, 26]; (2) the mutation was located in the transmembrane region of MT-CYB and altered an amino acid (L230I) with a high interspecific amino acid CI of 97.7%, indicative of the functional importance of the residue; (3) nonhaplogroups were associated on Phylotree; (4) from all 3,155 damaging alleles annotated in the UniProt database causing human Mendelian diseases and affecting protein stability or function, and from 6,321 differences between human proteins and their closely related mammalian homologues, it was assumed that the mutation was nondamaging. The second pair, HumVar, consists of all the 13,032 human disease-causing mutations from UniProt and 8,946 human nonsynonymous single-nucleotide polymorphisms (nsSNPs), and predicting the mutation as possibly damaging with a score of 0.563. All these findings indicated that the novel mutation had an effect on the mitochondrial oxidative metabolism and cellular physiology. 

Generally, missense mutations in MT-CYB may impair assembly or stability of the complex, leading to a dramatic decrease in the levels of assembled enzyme, or they may alter the catalytic activity of the complex with little effect on its assembly [27]. Several pathogenic variants in the MT-CYB gene have been previously reported linked to CMPs [9, 10]. One mutation that substituted a glycine by an aspartate residue at position 251 of cytochrome b (G251D) was associated with histiocytoid CMP [10]. This mutation was located in the intermembrane space loop connecting the fifth and sixth transmembrane segments. Analyses on mitochondria purified from patients' hearts have detected a defect in the succinate cytochrome c oxidoreductase activity and in the cytochrome b assembly, showing the importance of residue 251 for bc1 (complex III) function. A finding which challenges the high conservation of the m.15434C>A variant is the presence of the polymorphism m.15434C>T (p.230L>F) in two individuals and the fact that phenylalanine is not much different from isoleucine [28]. 

In conclusion, the present study implies that the MT-CYB gene mutation L230I plays a pathogenetic role in the development of primary CMPs. The novel potentially deleterious MT-CYB gene mutation L230I has been described for the first time in a patient with primary dCMP. However, prospective studies on yeast or transgenic mice with defined mutation are warranted to study the impact and contribution of this mutation on the pathophysiology of dCMP. 

## Figures and Tables

**Table 1:** Clinical cardiologic and echocardiographic characteristics of patients with dCMP and hCMP.

Type of CMP	hCMP	dCMP
Number of patients (*n*)	30	40
Mean ± 2SD age (years)	41.87 ± 19.13	39.3 ± 15.2
Mean NYHA class III and IV (%)	50%	72%
Mean LVDD ± SD on echocardiography (mm)	49.19 ± 11.76	67.05 ± 8.64
Mean ejection fraction (EF) ± SD on ventriculography (%)	34.73 ± 8.16	19.72 ± 6.83
Mean septal wall thickness (mm)	17.77 ± 3.20	9.46 ± 2.00
Mean posterior wall thickness (mm)	14.83 ± 2.65	9.36 ± 1.95

**Table 2:** Non-synonymous MT-CYB mutations identified in patients with CMPs.

Variant	dCMP (*n* = 40)	hCMP (*n* = 30)	Co (*n* = 50)	Amino acid position, physicochemical property	CI	Haplogroup specific variant	Status
C14766T	30/40	7/30	26/50	T7I, polar (T) to non polar (I)	0.279	B2a1a, M2b, E1a1, HV	Polymorphism
A14769G	2/40	0/30	0/50	N8S, medium, polar (N) to small, polar (S)	0.581	L1b, L3f1b, L3f1b	Polymorphism
T14798C	1/40	0/30	5/50	F18L, large, aromatic (F) to medium, hydrophobic (L)	0.581	J1c, T2g, K, L1c6	Polymorphism
A14927G	1/40	0/30	0/50	T61A, medium, polar (T) to small, hydrophobic (A)	0.581	D4b1a, D5c, U6a1	Polymorphism
G15257A	1/40	0/30	0/50	D171N, medium, acidic (D) to medium, polar (N)	**0.860**	K1b1a, M71a1, J2	Possibly deleterious
G15314A	1/40	0/30	1/50	A190T, small, hydrophobic (A) to medium, polar (T)	0.326	R9b2, L3j, L3k, M38, D4a1a1, P4a, L3j,	Polymorphism
A15326G	8/40	5/30	49/50	T194A, medium, polar (T), to small, hydrophobic (A)	0.442	Z3a1, H2a2a, R7b1a, R8a	Polymorphism
C15434A	1/40	0/30	0/50	L230I, both residues are medium and hydrophobic.	**0.977**	Non-haplogroup variants	Novel missense mutation
C15452A	2/40	0/30	6/50	L236I, similar physicochemical property, both residues are medium size and hydrophobic	**0.744**	JT	Polymorphism
G15803A	1/40	0/30	0/50	V353M, similar physicochemical property, both residues are medium size and hydrophobic	0.395	L2a2a	Polymorphism
G15884C	1/40	0/30	0/50	A380P, small, hydrophobic (A) to medium, hydrophobic (P)	0.047	L0F2b	Polymorphism

The tool PolyPhen-2 (http://genetics.bwh.harvard.edu/pph2/) was used for predicting the damaging effect of a missense mutation. The conservation index (CI) was computed by using MitoTool (http://www.mitotool.org/). The presence of the mutation as a haplogroup-specific variant was scored relative to the available global mtDNA phylogenetic tree by using Phylotree (http://phylotree.org/tree/main.htm). Co: controls.

**Table 3:** Silent mutations (synonymous single nucleotide polymorphisms) in the mtDNA cytochrome b gene (MT-CYB) identified in patients with CMP.

Variants	dCMP *n* = 40	hCMP *n* = 30	Controls *n* = 50	Amino acid position	Status
T14935C	1	0	0	F63F	Polymorphism
T15344C	1	0	0	L200L	Polymorphism
C15632T	1	0	0	L296L	Polymorphism
A15679G	1	1	0	K311K	Polymorphism
A15799G	1	0	0	G351G	Polymorphism
T14783C	0	0	2	L13L	Polymorphism
C14905A	1	1	1	M53M	Polymorphism
G15043A	1	1	4	G99G	Polymorphism
T15115C	1	0	0	T123T	Polymorphism
G15148A	1	0	0	P134P	Polymorphism
A15244G	1	0	1	G166G	Polymorphism
G15301A	1	1	4	L185L	Polymorphism
T15454C	0	1	0	L236L	Polymorphism
T15514C	1	1	0	Y256Y	Polymorphism
T15530C	1	0	0	L262L	Polymorphism
T15787C	1	0	0	F347F	Polymorphism

The 16 variants were interpreted as synonymous mutations, which do not cause any amino acid change, and were reported as single nucleotide polymorphisms in MITOMAP: A Human Mitochondrial Genome Database. (http://www.mitomap.org/, 2011).
